# Hot Springs

**Published:** 1896-01

**Authors:** E. T. Cook

**Affiliations:** Hot Springs, Ark.


### Correspondence.

### Hot Springs.

HOT SPRINGS, ARK., November 14, 1895.

*Editor Texas Medical Journal:*

It occurs to me, since I think of it, that I have never seen in a medical print a write-up of this great health resort. The fact that it stands pre-eminent as a place to which invalids go for certain kinds of troubles, seems to be admitted by all, but the peculiar virtue of the waters, and the vast number of people who are benefited thereby, seems to be lost sight of by the general public, and the medical profession in particular. For this reason I send a few dots to the JOURNAL, giving things as I see them, and impressions that have forced themselves upon me since I have been here. Hot Springs, to the medical profession, has borne an unenviable reputation for years, from the fact that a horde of unprincipled men, called "doctor drummers," have been wont to pounce upon the unsophisticated visitor and carry him, "whether or no," bodily to some other physician, other than the one to whom he wished to go. To accomplish his end, this same Mr. Drummer stopped at nothing. He would even report the M. D., to whom our hayseed friend was going, as dead, and with tears flowing, amid his sobs, he would announce the hour of the said

M. D.'s burial. If necessary to carry his point, and there was enough money in it, he would even get up a funeral procession for the occasion, to properly impress his "fruit," as he facetiously called his victim. Of course he would have his victim on some convenient corner where he could see the hearse pass. It is said, as an actual fact, that Allen Pinkerton, the great detective, was once the victim of these sharpers, and in recognition of their shrewdness gave a big supper in their honor, on which occasion he frankly admitted that they had buncoed him, and they were the only men on earth who had been smart enough to do so, and as a consequence he had no hesitancy in pronouncing them the "shrewdest rascals on God's green earth." It is really beyond belief to hear of the extremes to which these men went in their nefarious schemes. So vile and soulless did these outrages become that the legislature of the State tried to frame laws that would put an end to them. But it was of no avail. Men who were shrewd enough to work the schemes these men did, could easily find a way to beat the law. At length, but after the fair name of Hot Springs had suffered much injury, the physicians of the place got together and formed what they termed the "anti-drumming association." By iron-clad pledges and strict methods they have about succeeded in driving the drummer from the Springs, and at the present time drumming, if carried on at all, is done so so secretly, that it is not apparent on the surface. This is a God-send to the place, the physician and to legitimate medicine. Here, where nature has done so much in constructing what undoubtedly is the greatest health resort on earth; here, where, when one sees the wonderful results of the use of these waters, he is almost constrained to think that the Great Healer has established His Own sanitarium, it was, indeed, an outrage that the poor invalid, often reduced to his last penny, should be robbed and deprived of his last means of succor. It is different now, and the man of ordinary intelligence, if properly instructed, can, without difficulty, get to the physician he wants, or to the hotel at which he wishes to stop. Good lodgings can be gotten here for one dollar per week, and wholesome meals are served, at many restaurants, for from 12½ to 25 cents. If, however, the visitor wants comfort, or even elegance, he can find as magnificent accommodations as the continent affords, for here in Hot Springs are some of the most palatial hotels to be found in America. It is the place where the poor may find the most reasonable accommodation, and the rich may revel in the gaudiest of luxury.

As to the virtue that exists in the waters, while analyses show various chemical constituents, it is about accepted now that in the caloric lies the virtue. The eliminative power is so great that the patient can take enormous doses of medicine. In fact, what would be a toxic dose away from here, can be given with impunity, the skin eliminating chiefly, while the kidneys are very active and assist in getting clear of the overplus. The bath houses, in many instances, are elegant, and are supplied with all the conveniences for giving any kind of bath. Some are fitted with gymnasiums and electrical appliances. The charges at the bath houses vary, according to the purse or desires of the bather. Good, clean baths, with attendance, are given as low as $5.50 per course of twenty-one baths, or the more exquisite, can pay, if they choose, $15.00, and thereby bathe luxuriously. The physicians, in ordinary cases, treat for $25.00 per course of three weeks, and for surgery, charge according to the ability of the patient to pay.

By the way, some grand fees have been gotten here; but times are changing, and the Hot Springs doctor is no longer making the princely income that he once did. On the whole, this is a great place for invalids, and on account of its close proximity to Texas, is liberally patronized by Texans. The idea prevails that a course is essential here to all who contract syphilis. Of course this is fallacious, but great good is accomplished here to the patient from the fact that the physician holds his patient so well in hand, and when he comes he obeys to the letter the minutest instructions of his doctor. Away from here, he will not do this. After the primary sore disappears, he will cease to take his medicine. Here he will sit up at night to take it, if so instructed to do. Herein lies the chief advantage of a pilgrimage to Hot Springs. The idea that physicians in other places are not as competent to treat successfully this disease as those located here, is the height of foolishness, and if the patient could be taught to obey his doctor, time and money would be saved. Of course, it goes without saying that the hot water assists greatly in quickly purging the system of the disease.

The United States government is spending large sums here in beautifying its grounds. The United States Hospital on the mountain top is a marvel of beauty, presided over by an accomplished surgeon detailed from the regular army for this specific purpose. When the government works are completed, it will well be worth a trip here to see the place. Quaint and romantic sit the different structures on the mountains, and as the varied colors of the foliage come to view, now that they are in the "seer and yellow leaf," one can not but be impressed with their dazzling beauty, and feel that poetic thought that impresses on us the fact that "God made all things beautiful."

E. T. COOK.

